# CT Angiography Assessment of Dorsal Pancreatic Artery and Intrapancreatic Arcade Anatomy: Impact on Whipple Surgery Outcomes

**DOI:** 10.3390/tomography11010009

**Published:** 2025-01-14

**Authors:** Gorkem Ozdemir, Tolga Olmez, Okan Dilek, Berkay Eyi, Alper Sozutek, Ahmet Seker

**Affiliations:** 1 Department of Gastroenterological Surgery, Adana City Training and Research Hospital, 01370 Adana, Turkey; 2 Department of Radiology, Adana City Training and Research Hospital, 01370 Adana, Turkey; dr.okandilek@gmail.com (O.D.);

**Keywords:** dorsal pancreatic artery, complication, intrapancreatic arcade, pancreaticoduodenectomy, whipple procedure, computed tomography, CT Angiography

## Abstract

Background/Objectives: The aim was to investigate the association between variations in the dorsal pancreatic artery (DPA) and intrapancreatic arcade anatomy with Whipple procedure outcomes and postoperative complications. Methods: This retrospective study was conducted with 362 patients who underwent a Whipple procedure at the Department of Gastroenterological Surgery of Adana City Training and Research Hospital between January 2018 and April 2024. All data collected from medical records were compared and statistically analyzed according to the patients’ survival status and arcade subtypes. Results: After excluding cases that did not meet the study criteria, a total of 284 patients were included in the study. DPA was visualized in 55.98% (159/284) of patients, while the intrapancreatic arcade was observed in 25% (71/284). The most common origin of the DPA was the splenic artery in 69.2% (n = 110) of patients, followed by the superior mesenteric artery in 17.6% (n = 28). The frequency of intrapancreatic arcade anatomy variations was as follows: type 1: 28.2% (n = 20), type 2: 49.3% (n = 35) and type 3: 22.5% (n = 16). Arcade type 4 anatomy was not detected. Postoperative pancreatic fistula (POPF) complication was found to be statistically significantly higher in patients with type 3 anatomy (*p* = 0.042). The 90-day mortality and long-term mortality rates did not differ among the groups based on the variations in both DPA and intrapancreatic arcade anatomy types. Conclusions: Patients with intrapancreatic arcade type 3 anatomy had a higher risk of POPF complications. Determination of preoperative arcade type by computed tomography (CT) angiography may help to predict the risk of POPF.

## 1. Introduction

Pancreaticoduodenectomy (PD), known as the Whipple procedure, is a complex surgical procedure usually performed for malignant tumors of the pancreas and distal bile duct [1,2]. This procedure, which causes significant complications, is known to be associated with postoperative morbidity ranging from 30% to 60% [3,4]. Complications that arise due to the Whipple procedure include pancreatic leak or postoperative pancreatic fistula (POPF), bile leak, intra-abdominal abscess, postoperative bleeding requiring blood transfusion, and infection [5,6].

The pancreas receives blood supply from various sources, primarily the celiac trunk and the superior mesenteric artery (SMA). The splenic artery (SA) plays a crucial role in supplying the body and tail of the pancreas, with the dorsal pancreatic artery (DPA) being a key contributor [7,8]. The intrapancreatic arcade exhibits significant variability among individuals. Understanding the anatomical variations is crucial for surgeons and interventional radiologists to effectively plan and execute pancreatic surgeries [9].

Variations in the branches of the celiac artery are common and can significantly increase the complexity of the Whipple procedure [10]. Aberrant hepatic artery variation does not seem to influence the morbidity, mortality and tumor resection margins in patients undergoing Whipple’s procedure [11,12]. Crocetti et al. found that the presence of a replaced right hepatic artery (RHA) was associated with increased blood loss and a longer Intensive Care Unit stay [13]. However, it did not significantly impact oncological outcomes, surgical morbidity or long-term overall survival compared to standard PD procedures. Perwaiz et al. and Rammohan et al. reported similar findings, with the exception of Rammohan et al., who did not observe significant differences in blood loss between patients with and without RHA [14,15]. On the other hand, hepatic artery injury during resection predisposes to various complications including liver necrosis and liver abscess in PD surgery [16].

While variations in the hepatic, celiac and superior mesenteric arteries in patients undergoing PD have been extensively studied, variations in the pancreatic arterial supply have received comparatively less attention in the literature [10,11,17]. Therefore, this study aimed to investigate the association between variations in the DPA and intrapancreatic arcade anatomy with Whipple procedure outcomes and postoperative complications.

## 2. Materials and Methods

### 2.1. Patients and Image Interpretation

Between January 2018 and April 2024, 362 patients who underwent Whipple surgery in our clinic were retrospectively included in the study. One of the radiologists screened all cases for image quality, excluding patients with respiratory or motion artifacts or inadequate phase imaging. Of 362 patients, 37 patients without contrast administration and 41 patients with poor image quality were excluded.

Multidetector contrast-enhanced computed tomography (CT) (triple-phase CT abdomen) scans of the remaining 284 patients were retrospectively evaluated. All images were interpreted at a Philips Intellispace Workstation. All patients were anonymized and CT scans were analyzed by a radiologist who specialized in abdominal imaging and a gastroenterological surgeon with consensus. Readers were blinded to other imaging studies of the same patient. When disagreements arose regarding image interpretation, consensus was reached through additional review sessions involving both the radiologist and the surgeon.

During image analysis, 3D reconstructions were employed, including multiplanar reconstruction, maximum intensity projection, and volume rendering. These techniques were actively utilized throughout the interpretation phase.

**Figure 1:**
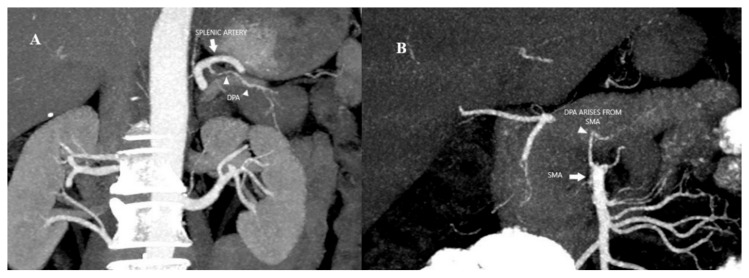
(**A**) Coronal MIP image of arterial phase of CECT abdomen shows DPA (arrowhead) arising from splenic artery (arrow). (**B**) Coronal MIP image of arterial phase of CECT abdomen shows DPA (arrowhead) arising from superior mesenteric artery (arrow).

**Figure 2:**
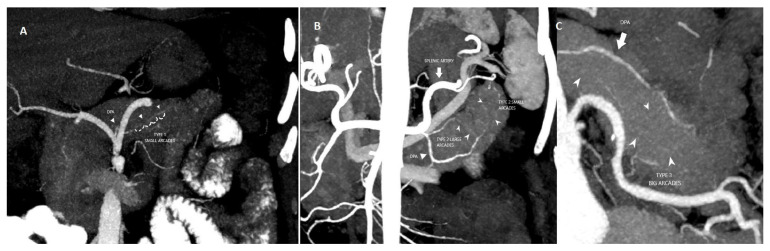
(**A**) Coronal MIP image shows type I pancreatic arcade with multiple small branches. (**B**) Coronal MIP image shows type II pancreatic arcade with small and large branches. (**C**) Coronal MIP image shows type III large pancreatic arcades.

Readers were tasked with assessing the following variables: (a) origin of the DPA and (b) intrapancreatic arcade classification type. The origin of the DPA was evaluated (Figure 1). Previously, the anatomy of the intrapancreatic arcade has been categorized into four types: type 1 (minor arcades), type 2 (minor and major arcades), type 3 (major arcades) and type 4 (straight branches) according to Roman Ramos et al. [18]. The intrapancreatic arcade anatomy was grouped according to this classification (Figure 2)

All patients underwent the Whipple procedure by the same surgical team.

### 2.2. Data Collection

A retrospective analysis was conducted on consecutive pancreaticoduodenectomies performed at our clinic from January 2018 to April 2024. The patient records were examined, and the following data were collected for each patient: age, gender, indication for the surgery, tumor location, tumor diameter, the presence of postoperative pancreatic fistula, biliary fistula, per-operative red blood cell transfusion, length of hospitalization, postoperative complications and mortality rates.

In this retrospective study, demographic, operative and postoperative data of the patients were collected using patient records. All data of the patients with no missing information in the data records were compared with DPA variations and the intrapancreatic arcade subtypes. The postoperative complications were graded by the Clavien–Dindo classification. The classification is a widely used system for grading postoperative complications [19]. It categorizes complications based on their severity, ranging from minor to major. It can be summarized as follows:

Grade I: minor complications that require pharmacological treatment or minor interventions. Grade II: complications requiring invasive intervention, such as drainage or endoscopic procedures. Grade IIIa: serious complications requiring non-operative intervention. Grade IIIb: serious complications requiring operative, endoscopic, or radiological intervention. Grade IV: life-threatening complications. Grade V: death.

### 2.3. CT Angiography Protocol

All CT scans were performed using a 128-detector multi-detector CT unit (Philips Ingenuity 128, Eindhoven, The Netherlands). The technical parameters were 120 kvP, 200–400 mAs, rotation time 0.42 s, and slice thickness 1 mm for all phases. Contrast-enhanced scanning was performed by injections of saline, non-ionic iodinated contrast media and, finally, 20 cc saline, in sequential order, and by means of an automatic infusion pump. The contrast media dose was 2.5–4.0 mL/kg. Following the injection of the contrast material, pancreatic, portal and late phase scans were obtained at 35 s, 60 s and 120 s, sequentially. All scans were obtained with the patient at breath-hold full inspiration.

### 2.4. Statistical Analysis

Descriptive statistics are summarized as counts and percentages for categorical variables, mean ± standard deviations or median for continuous variables. The differences between groups in terms of categorical variables were compared using the Chi-square test. If expected counts were less than five in cells, an exact test was performed. The dual category was excluded from the analysis due to low sample size (n = 2) while comparing DPA variation categories. The difference between two groups for normally distributed continuous variables was evaluated by Student’s t test. The Mann–Whitney U test was used to test the difference between two groups in non-normally distributed continuous variables. For intrapancreatic arcade categories, the Kruskal–Wallis test was performed while comparing the length of hospitalization. A *p*-value less than 0.05 was considered statistically significant. IBM SPSS 26 (Armonk, NY, USA: IBM Corp) was used for statistical analysis.

## 3. Results

Our study population consisted of 284 patients, of whom 150 were female and 134 were male. DPA was visualized in 159 (55.98%) of the 284 patients, while it was not visualized in the remaining 125 (44.02%).

According to the origin distribution of DPA, SA was the most common origin in 69.2% (n = 110) of the patients, the other origins were common hepatic artery (CHA) 4.4% (n = 7), gastroduodenal artery 3.8% (n = 6), SMA 17.6% (n = 28), celiac artery 3.8% (n = 6), dual SA–gastroduodenal arteries 0.6% (n = 1) and dual SA-SMA arteries 0.6% (n = 1) (Figure 1).

In the DPA group, the mean age of the patients was 60.9 ± 11.7 years and the median age was 62 (IQR: 54–71) years. The indications for Whipple surgery were as follows: malignant lesion (n = 121), pancreatitis (n = 27), pre-malignant lesions including serous cystadenoma and intraductal papillary mucinous neoplasm (IPMN) (n= 9) and trauma (n = 2). The site of tumor was ampulla in 31.4% (n = 63), pancreas in 31.4% (n = 38), choledoc in 7.4% (n = 9), duodenum in 6.7% (n = 8), pylorus in 1.7% (n = 2) and colon in 0.8% (n = 1) of the patients. The mean tumor diameter was 2.8 ± 1.8 cm and the median diameter was 2.5 cm (IQR: 1.5–3.5). The mean follow-up time in this group was 27.60 ± 19.88 months. The 90-day mortality and long-term mortality rates did not differ among the groups (*p* ≥ 0.05) (Table 1).

**Figure 3:**
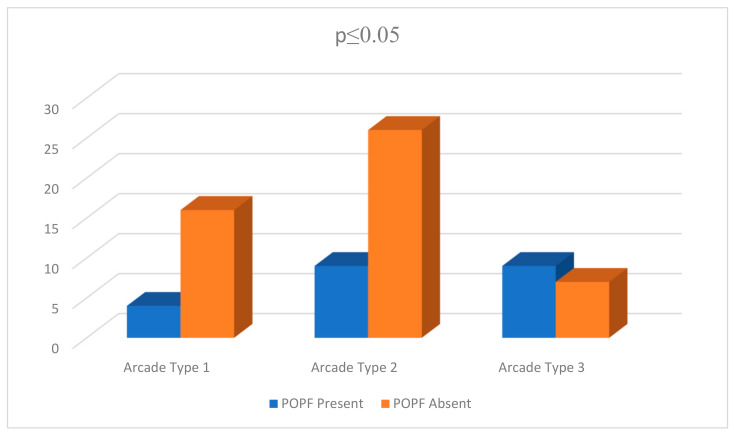
Comparison of POPF rates according to the different types of intrapancreatic arcade anatomy.

The intrapancreatic arcade was observed in 25% (71/284) of patients. The distribution of arcade anatomy was as follows: 28.2% (n = 20) arcade type 1, 49.3% (n = 35) arcade type 2 and 22.5% (n = 16) arcade type 3 (Figure 2). Arcade type 4 anatomy was not detected. The mean follow-up time in this group was 31.85 ±19.59 months. The 90-day mortality and long-term mortality rates did not differ among the groups in this group (*p* ≥ 0.05). The presence of POPF was significantly different between the groups (*p* = 0.042); a higher proportion of POPF was observed in group 3 (Table 2) (Figure 3). There were no significant differences between the two groups with regard to gender distribution, the presence of systemic disease, or the length of hospitalization (Table 2).

When we grouped the data according to the variations in intrapancreatic arcade anatomy, we did not observe any differences between groups in terms of indication for surgery (benign or malignant), tumor location and tumor size (*p* ≥ 0.05). These findings reveal that there are similar distributions among the arcade categories in terms of many clinical and demographic variables, but there is a significant difference in the presence of POPF (Table 2).

Table 2 presents the comparison of post-Whipple surgery outcomes according to intrapancreatic arcade variations.

## 4. Discussion

Although morbidity and mortality in the Whipple procedure have improved with advances in surgical techniques in recent years, the prevalence of postoperative complications remains high [20]. Identification of the mechanisms that may cause complications is important in reducing postoperative risks. Our study showed that POPF complication was significantly higher in patients with type 3 intrapancreatic arcade.

A meta-analysis focusing on the influence of aberrant peripancreatic arterial anatomy on outcomes of PD showed that the most common abnormalities of the hepatic vasculature include a replaced RHA, replaced left hepatic artery (LHA), and accessory RHA or LHA, as well as arcuate ligament syndrome (causing celiac artery stenosis), which are also linked to complications following pancreatic surgery [21]. Damage to the biliary branches of the hepatic arteries increases the risk of postoperative biliary anastomotic leak [21]. Furthermore, aberrant arterial anatomy in patients undergoing pancreatic surgery increases the risk of damage to the blood vessels supplying the liver, which can result in unexpected bleeding (during or after surgery) or insufficient blood flow to the liver (ischemia) [22].

In a cadaveric study, a rare case with a replaced RHA and two dorsal pancreatic arteries was reported. In that case, the right DPA originated from the gastroduodenal artery and the left DPA originated directly from the CHA [23].

Two studies in the literature confirm the high variability in the origin and number of pancreaticoduodenal arterial arcades [24,25]. Both studies found multiple arcades to be common, with Szuák et al. reporting a slightly higher frequency (36%) compared to Macchi et al. (23.7%). Szuák et al. measured arcade diameters on casts, while Macchi et al. measured vessel diameters at their origins, leading to some differences in the reported values. Szuák et al. found that in terms of the origin of the superior pancreaticoduodenal artery, only 10% was from the gastroduodenal artery, whereas the inferior pancreaticoduodenal artery was found in 80% of cases; these data are comparable to those reported in Macchi et al.’s study on living patients (7.8% and 71.4%, respectively) [24,25].

Anatomical evaluation of the DPA is important when performing the Whipple procedure. The DPA originates from many sources, including the SA, CHA, celiac artery, SMA and other vessels [26]. Studies report that the origin of DPA is SA in 38.5–46.1% of cases, hepatic artery in 15.4–25.7% of cases, celiac artery in 7.7–8.6%, jejunal artery or middle colic artery in 5.7%, CHA in 7.7% and right gastroepiploic artery in 7.7% [27].

In our study, DPA originated from SA in 62.8% of the patients and from SMA in 17.6%. No statistically significant difference was observed in terms of origin between surviving and deceased patients. The distribution of intrapancreatic arcade types was type 1 (28.2%), type 2 (49.3%) and type 3 (22.5%), while type 4 was not detected in any patient. There was no statistically significant difference between arcade types and gender, biliary fistula, Clavien–Dindo classification, per-operative RBC transfusion, or the presence of systemic disease, but there was a statistically significant difference in terms of POPF complication.

Pancreatic fistula is a mortal complication affecting up to 30% of patients undergoing the Whipple procedure [28,29]. Various studies have been conducted aiming to predict fatal complications such as POPF before PD [29,30,31]. Kolbinger et al. emphasized in their study that risk factors can be determined by preoperative contrast-enhanced CT imaging [31]. Callery et al. reported that anatomic features such as small pancreatic duct diameter and soft pancreatic tissue as well as excessive intraoperative blood loss are risk factors for POPF [29]. Some previous studies have reported that high blood loss increases the risk of POPF and is a key component contributing to up to 30% of POPF development [29,32]. Casciani et al. emphasized that excessive intraoperative blood loss is a risk factor for POPF [33]. All of these studies reveal a relationship between blood supply and POPF development. In our study, we investigated the risk of POPF development according to the variations in DPA and intrapancreatic arcade anatomy subtypes different from the literature. Interestingly, POPF complications were significantly higher in patients with arcade type 3 subtype. The reduced number of intrapancreatic collaterals to other types in type 3 variation can negatively impact residual organ perfusion, resulting in decreased tissue perfusion. The increased local ischemia at the cut surface contributes to a higher risk of pancreatic leakage.

In some previous studies, ligation of the DPA has been reported to reduce bleeding during DP. Jiang et al. reported that ligation of DPA before dissection of the uncinate process improved resection time and blood loss in laparoscopic PD [34]. The right branch of the DPA supplying the pancreatic head is considered as one of the efferent arteries of the pancreatic head [26]. Studies have focused on the risk of intraoperative hemorrhage but have not sufficiently focused on the postoperative perfusional risks of the pancreas. In DPA arcade type 3, vascularization is provided only by the long branches of the SA [18]. We predict that the less branching in type 3 compared to other types may lead to insufficient postoperative pancreas vascularization. Considering the relationship between blood loss and pancreatic fistula, we think that malperfusion may indirectly lead to POPF development.

In the study conducted by Sharma S. et al., DPA was visualized in 65.3% of the patients and the intrapancreatic arcade was visualized in 25% of the patients with multidetector CT [9]. Similarly, in our study, DPA was visualized in 55.98% of patients, while the intrapancreatic arcade was observed in 25% patients. Additionally, the literature reports varying detection rates for the DPA (65.4–94%) [25,35,36]. During anatomical dissection, the DPA can be identified in 88.8% of cases [37]. This also supports the fact that its radiological evaluation is partially difficult. In our study, it could be visualized in approximately 56% of cases. Despite advanced technical developments in CT technology, we could evaluate the intrapancreatic arcade anatomy of the pancreas in very few cases. It is important to evaluate the variations in arcade anatomy with more appropriate techniques.

Whipple surgery is most commonly performed for malignant neoplasms, such as pancreatic head cancers. If preoperative CT angiography becomes a standard procedure before this surgery, this will enable an evaluation of the relationship between the mass and the blood vessels, thereby providing a better assessment of resectability. CT angiography is a more cost-effective and accessible imaging modality compared to MRI. This enables the identification of inoperable patients beforehand, preventing unnecessary surgical interventions. By evaluating both vascular variations and the relationship between the tumor and the vessels, surgical complications can be reduced.

Our study demonstrated that collateral and intrapancreatic variations may effect post-surgical complications. We think that gentle dissection and gentle transection during surgery will be beneficial in reducing complications, particularly the pancreatic fistula.

## 5. Conclusions

The risk of pancreatic fistula development after the Whipple procedure in patients with intrapancreatic arcade type 3 anatomy was statistically higher than other types. Determining the type of arcade anatomy preoperatively with CT may help surgeons predict the risk of POPF. Future studies can explain the relationships between arcade type and postoperative complications in detail.

## Figures and Tables

**Table 1:** Comparisons of Whipple surgery outcomes according to DPA variations.

	The DPA Variations						
	SA	CHA	GD	SMA	CELIAK	DUAL	*p Value* *
Frequency n (%)	110 (69.2%)	7 (4.4%)	6 (3.8%)	28 (17.6%)	6 (3.8%)	2 (1.2%)	
Gender (F/M)	60/50	5/2	4/2	15/13	4/2	1/1	0.873
POPF (+/−)	40/70	0/7	2/4	13/15	1/5	1/1	0.172
Biliary fistula (+/−)	6/104	1/6	0/6	1/27	0/6	0/2	0.777
Per-operative RBC Transfusion (Units) (0/1/2)	75/23/12	3/2/2	6/0/0	20/6/12	4/2/0	2/0/0	0.075
90-day mortality (+/−)	9/101	0/7	0/6	1/27	1/5	0/2	0.670
CLAVIEN–DINDO 1–2/3/4/5	56/83/11/9	6/1/0/0	3/2/1/0	15/10/2/1	3/2/0/1	1/1/0/0	0.259
Systemic disease (+/−)	64/46	2/5	3/3	15/13	1/5	0/2	0.206
Length of hospitalization median (IQR) days	12 (2–45)	8 (6–23)	10.5 (8–13)	12 (7–47)	19.5 (7–38)	11.5 (8–15)	0.167
Long-term mortality (+/−)	39/71	1/6	1/5	8/20	4/2	0/2	0.314

RBC: red blood cell, POPF: postoperative pancreatic fistula, SA: splenic artery, CHA: common hepatic artery, GD: gastroduodenal artery, SMA: superior mesenteric artery. * The dual category was excluded from the analysis due to low sample size (n = 2). For categorical data, Fisher’s exact test was performed.

**Table 2:** Comparison of post-Whipple surgery outcomes according to intrapancreatic arcade variations.

	Arcade Classification			*p Value*
	Type 1 (n = 20)	Type 2 (n = 35)	Type 3 (n = 16)	
**Gender (F/M)**	12/8	23/12	10/6	0.948
**POPF (+/−)**	4/16	9/26	9/7	0.042
**Biliary fistula (+/−)**	2/18	3/32	1/15	0.999
**Per-operative RBC Transfusion (Units) (0/1/2)**	18/1/1	23/8/4	11/3/2	0.376
**90-day mortality**	3/17	9/26	2/14	0.577
**CLAVIEN–DINDO 1–2/3/4/5**	10/4/3/3	19/10/3/3	8/3/3/2	0.887
**Systemic disease (+/−)**	7/13	12/23	5/11	0.969
**Length of hospitalization (Median)**	9 (6–17)	11 (7–20)	9 (6–19)	0.131
**90-day mortality (ex/alive)**	3/17	3/32	2/14	0.705
**Long-term follow-up (ex/alive)**	9/11	7/28	3/13	0.113

RBC: red blood cell.

## Data Availability

Data are available from the corresponding author upon request.
